# Cost-Effectiveness Analysis of Imaging Modalities for Breast Cancer Surveillance Among *BRCA1/2* Mutation Carriers: A Systematic Review

**DOI:** 10.3389/fonc.2021.763161

**Published:** 2022-01-10

**Authors:** Jiaxin Li, Ziqi Jia, Menglu Zhang, Gang Liu, Zeyu Xing, Xin Wang, Xin Huang, Kexin Feng, Jiang Wu, Wenyan Wang, Jie Wang, Jiaqi Liu, Xiang Wang

**Affiliations:** ^1^ Department of Breast Surgical Oncology, National Cancer Center/National Clinical Research Center for Cancer/Cancer Hospital, Chinese Academy of Medical Sciences and Peking Union Medical College, Beijing, China; ^2^ Department of Breast Surgery, Peking Union Medical College Hospital, Peking Union Medical College and Chinese Academy of Medical Sciences, Beijing, China; ^3^ Department of Breast Surgery, Beijing Tiantan Hospital, Capital Medical University, Beijing, China; ^4^ Department of Ultrasound, National Cancer Center/National Clinical Research Center for Cancer/Cancer Hospital, Chinese Academy of Medical Sciences and Peking Union Medical College, Beijing, China

**Keywords:** breast cancer surveillance, *BRCA1/2*, mammography, cost-effectiveness, MRI

## Abstract

**Background:**

*BRCA1/2* mutation carriers are suggested with regular breast cancer surveillance screening strategies using mammography with supplementary MRI as an adjunct tool in Western countries. From a cost-effectiveness perspective, however, the benefits of screening modalities remain controversial among different mutated genes and screening schedules.

**Methods:**

We searched the MEDLINE/PubMed, Embase, Cochrane Library, Scopus, and Web of Science databases to collect and compare the results of different cost-effectiveness analyses. A simulated model was used to predict the impact of screening strategies in the target group on cost, life-year gained, quality-adjusted life years, and incremental cost-effectiveness ratio (ICER).

**Results:**

Nine cost-effectiveness studies were included. Combined mammography and MRI strategy is cost-effective in *BRCA1* mutation carriers for the middle-aged group (age 35 to 54). *BRCA2* mutation carriers are less likely to benefit from adjunct MRI screening, which implies that mammography alone would be sufficient from a cost-effectiveness perspective, regardless of dense breast cancer.

**Conclusions:**

Precision screening strategies among *BRCA1/2* mutation carriers should be conducted according to the acceptable ICER, i.e., a combination of mammography and MRI for *BRCA1* mutation carriers and mammography alone for *BRCA2* mutation carriers.

**Systematic Review Registration:**

PROSPERO, identifier CRD42020205471.

## Introduction

Currently, breast cancer is a common cancer worldwide (1). Women with germline mutations in cancer predisposition genes develop breast cancer with cumulative risks, e.g., 55% of *BRCA1* mutation carriers and 45% of *BRCA2* mutation carriers were 80 years old (2). In addition, women with a positive familial history and dense breasts were also characterized as high-risk women (3). These women, especially *BRCA1* mutation carriers, develop breast cancer at younger ages with an increased possibility of triple-negative breast cancer (4). Intensive breast screening modalities were more widely undertaken than bilateral prophylactic mastectomy (5). Thus, a regular screening regimen is necessary and important for *BRCA1/2* mutation carriers and high-risk women.

Currently, mammography is still the most widely used modality in Western countries, as it has been confirmed to reduce breast cancer mortality (6, 7). However, for *BRCA1/2* mutation carriers, it has been demonstrated that mammography has limited performance in cancer detection related to their benign appearance and high interval cancer rate (8). MRI serves as a diagnostic tool with the highest sensitivity, and it performs well on dense breasts (9). Considering that there is no radiation harm to patients, MRI is suggested to start at an early age. However, the high cost, increased false-positive rate, and unnecessary biopsies should not be neglected (10, 11). The combination of MRI and mammography would increase sensitivity compared with mammography alone among *BRCA1/2* mutation carriers (12). Although screening ultrasound has been confirmed to show comparable performance with improved cancer detection, it has exceeded the false-positive rate, resulting in the subsequent cost of more benign biopsies (13). In addition, ultrasound and clinical breast examination have not appeared to add addictive benefits for the screening regimen in Western countries (14, 15). These findings mainly focused on *BRCA1/2* mutation carriers. Women without high breast cancer risk could not gain an advantage when performing combined mammography and MRI (16).

Therefore, a combination of mammography and MRI is recommended. For *BRCA1/2* mutation carriers, the National Comprehensive Cancer Network (NCCN) recommends the combination of annual MRI from 25 to 75 years old and annual mammography from the age of 30 to 75 (17). Different guidelines vary in the start and terminal age of screening: the National Institute for Health and Care Excellence (NICE) recommends annual MRI for patients aged 30 to 49 years and annual mammography for patients aged 40 to 69 years (18). The European Society for Medical Oncology (ESMO) suggests annual MRI from age 25, and it proposes a combination of annual MRI with annual mammography from age 30 (19). Several factors influence adherence to regular screening regimens in *BRCA1/2* mutation carriers, including disease suffering load, patient awareness, education level, country development, access to MRI, and so forth. More importantly, the cost-effective intervention of different screening strategies for high-risk women carrying *BRCA1/2* mutations is still unclear.

Herein, we focused on cost-effectiveness benefits from screening combinations of MRI and mammography in comparison with mammography alone in *BRCA1/2* mutation carriers. Direct outcomes of lifetime cost, life-years gained (LYG), and quality-adjusted life years (QALYs) were collected to compare the incremental cost-effectiveness ratio (ICER) (20). In cost-effectiveness analysis, a cost-effective intervention was evaluated with ICER falling in a definitive threshold (21). This review may help health policymakers make informed, optimal, and unified decisions.

## Materials and Methods

### Study Design and Search Strategy

We developed a research question according to PICOT (Population, Intervention, Comparison, Outcome, Time): “Which screening strategy is more cost-effective comparing the combination of MRI and mammography with mammography alone among *BRCA1/2* mutation carriers from different ages?” (22). The PICOT question aided in the selection and evaluation of studies.

We conducted a systematic literature search for studies published from January 1990 to September 2020 in the following databases: MEDLINE/PubMed, EMBASE, Scopus, Cochrane Library, and Web of Science. We searched for key terms, including *BRCA1* and *BRCA2*, breast cancer, cost-effectiveness, and screening (**Supplementary Table S1**). We also screened eligible reviews to select relevant studies in reference lists. This review was conducted under PRISMA guidelines (23). The study was registered in the International Prospective Register of Systematic Reviews (PROSPERO, CRD42020205471).

Articles were screened by two researchers independently. An economic evaluation for cost-effectiveness analysis should meet the following criteria: i) it should be a cost-effectiveness analysis that had available outcomes of costs, LYG, QALYs, and ICER; ii) it should focus on the target population of *BRCA1/2* mutation carriers; and iii) it should include screening strategies of mammography, MRI, or the combination of the two. After screening titles and abstracts, full-text documents were acquired to select the final inclusive studies by the criteria above. Disagreements among the included articles were discussed to reach a consensus.

Mainly, we investigated cost-effectiveness analyses with sufficient evaluation outcomes such as cost, QALYs or LYGs, and ICER. The following types of studies were not included: a) studies that used the insufficient decision-analytic model to conduct cost-effectiveness analysis, b) studies that did not compare cost-effectiveness between a combination of MRI and mammography, c) studies that merely included patients with dense breasts, d) studies that conducted complicated comparisons between different screening strategies among nations, and e) studies that only involved high-risk women.

### Data Extraction and Quality Assessment

We used a standardized evidence table to collect definite information, which included model-related characteristics (author, country/region, target population, model type, perspective, type of cost, discount rate, currency, outcomes, sensitivity analysis, threshold). The cost-effectiveness (cost, QALYs, LYG, ICER) and description of the screening method were also evaluated. Cost-effectiveness analysis models were monitoring life cycles of the target population and comparing the consequence and cost in the procedure. ICER was calculated by comparing the incremental cost of two screening strategies divided by incremental effects. Incremental QALYs and LYGs were the incremental effects. In these model-based cost-effectiveness analyses, *BRCA1/2* mutation carriers were simulated female and hypothetical individuals. Among different studies, simulated *BRCA1/2* mutation carrier women were majorly introduced into the model from age 25 years with no breast cancer and prophylactic surgery history. Parameters including breast cancer incidence, the detection rate of screening surveillance, and other related parameters were extracted from national cancer databases and critical literature review.

We used the Consolidated Health Economic Evaluation Reporting Standards (CHEERS) quality assessment checklist to calculate the scores of the included studies (24).

Several reviews about cost-effectiveness analysis transformed into one unified currency; however, the evaluation of cost-effectiveness varies by region or country. As a result, we used the threshold mentioned in the previously selected study to make a further comparison.

## Results

### Study Selection

**Figure 1:**
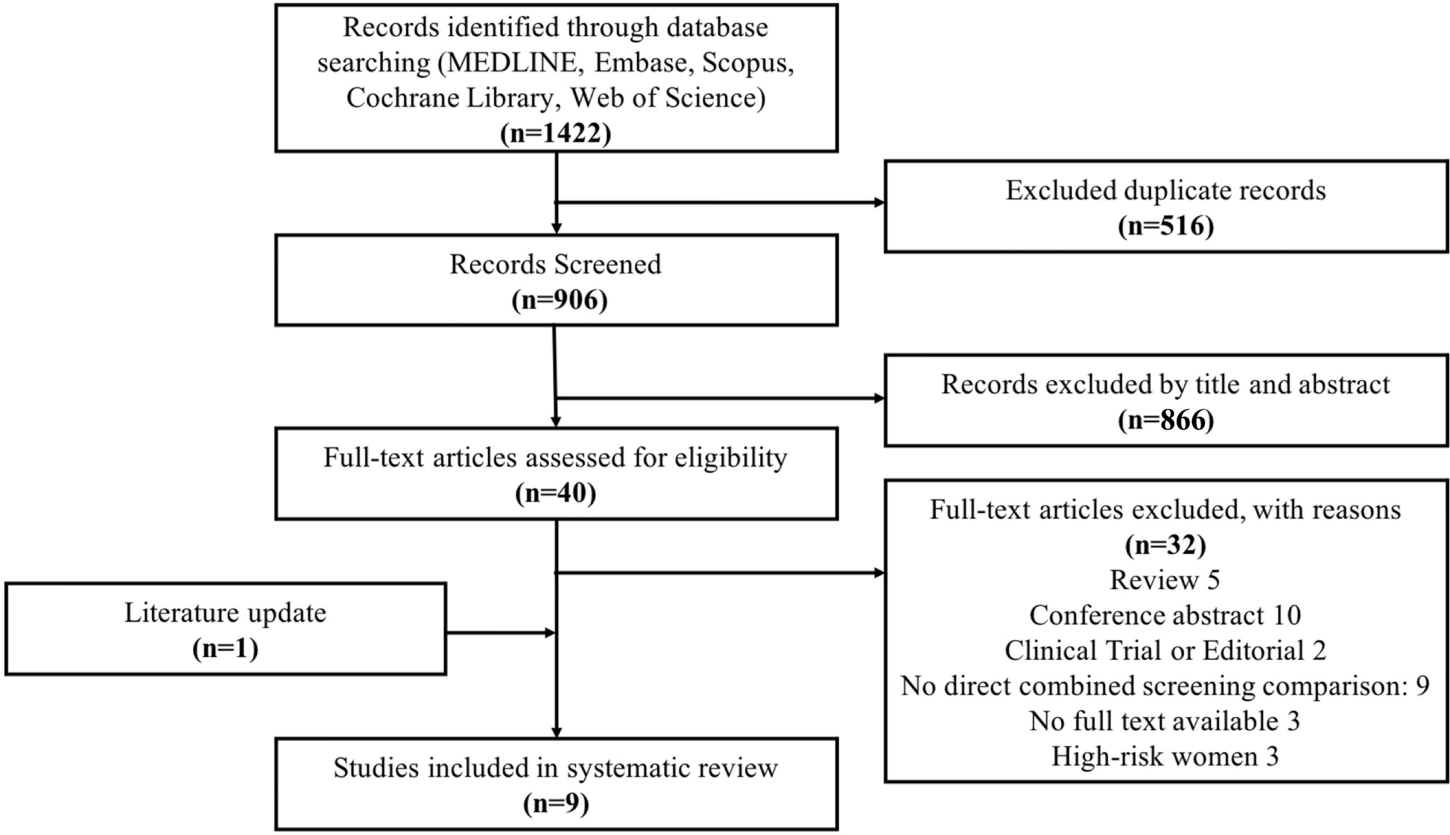
The PRISMA flow diagram for the study selection: search for cost-effectiveness analyses evaluating screening strategies among *BRCA1/2* mutation carriers and high-risk women. The exclusion criteria were as follows: review, conference abstract, clinical trial or editorial, no full text available, high-risk women, and no direct combined screening comparison (*N* = 9), which included studies not presenting a combination of MRI and mammography (*N* = 6), studies comparing the screening over countries (*N* = 1), studies involving patients with dense breast (*N* = 1), and studies with non-standard cost-effectiveness analysis (*N* = 1).

There were 1,422 references with 516 duplicates. We screened 906 references through titles and abstracts, and we selected 40 references for full-text investment, with eight (25–32) meeting the inclusion criteria. Eventually, after adding one (33) reference from the updated literature search, nine studies were included (**Figure 1**).

### Description of Studies: Key Characteristics

Selected studies covered a small number of countries, including five in the United States, one in the United Kingdom, two in the Netherlands, and one in Canada (**Table 1** and **Supplementary Tables S2**, **S4**). The models simulated women as the target population and reported three (26, 27, 29) in solely *BRCA*1 mutation carriers, four (25, 28, 30, 33) in both *BRCA1* and *BRCA2* mutation carriers, and two (31, 32) in discriminatory *BRCA1/2* mutation carriers. Among the nine selected studies, *BRCA* mutation carriers were simulated women cohorts with no breast cancer history or prophylactic surgery. Associated parameters in building cost-effectiveness analysis were originated from national databases and literature reviews. Eight studies used the model to simulate the screening process, except that one study did not mention the model type. All nine studies calculated the direct cost, including screening, treatment for breast cancer, and other costs related to disease. In particular, three studies involved indirect costs for loss of productivity and time. The discount rate mainly ranged from 3% to 3.5% using international data in nine studies, despite one also considering the discount rate by the nation. Furthermore, the outcomes measured QALYs gained in seven studies and LYGs in two to obtain ICER. ICER was calculated by comparing incremental cost divided by incremental effects (QALYs and LYGs) of the two strategies: one was the combination of mammography and adjunct MRI and the other was mammography alone. Threshold definition was not mentioned in four studies, and we assumed the threshold definition to be the same as the remaining studies for analysis.

**Table 1:** Information of cost-effectiveness analysis from the study selection.

Study: Author (year); Country/region	Target population	Model type; Perspective; Type of cost; Discount rate; Currency	Outcome measures	Sensitivity analyses	Threshold definition
Plevritis et al. (2006); USA (25)	*BRCA1* and *BRCA2* mutation carriers	Continuous time Monte Carlo; Societal; Direct and indirect; 3%; 2005 US dollars	QALYs Cost; ICER (cost/QALY gained)	One-way, multivariate sensitivity analysis	Cost-effectiveness threshold of $100,000 US dollars
Norman et al. (2007); UK (26)	*BRCA1* mutation carriers	Markov; National Health Service; Direct; 3.5%; 2006 UK pounds	QALY; Cost; ICER (cost/QALY gained)	Univariate sensitivity analysis and probabilistic sensitivity analysis	Cost-effectiveness threshold of £20,000 pounds
Lee et al. (2010); USA (27)	*BRCA1* mutation carriers	Markov Monte Carlo; Societal; Direct; 3%; 2007 US dollars	QALY; Cost; ICER (cost/QALY gained)	Univariate sensitivity analysis and multivariate sensitivity analysis	Cost-effectiveness threshold of $50,000–100,000 US dollars
Grann et al. (2011); USA (33)	*BRCA1* and *BRCA2* mutation carriers	Markov Monte Carlo; Societal; Direct and indirect; 3%; 2009 US dollars	QALY; Cost; ICER (cost/QALY gained)	Probabilistic sensitivity analysis	Threshold not reported Assumed as <$100,000
Cott et al. (2013); USA (28)	*BRCA1* and *BRCA2* mutation carriers	Markov Monte Carlo; Perspective not mentioned; Direct and indirect; 3%; 2010 US dollars	QALY Cost; ICER (cost/QALY gained)	Univariate sensitivity analysis; two-way, multiparameter sensitivity analysis	Threshold not reported Assumed as <$100,000
Obdeijn et al. (2016); Netherlands (29)	*BRCA1* mutation carriers	Microsimulation; Healthcare system; Direct; 3.5%; Not clearly mentioned Euros	LYG; Cost; ICER (cost/LYG)	Univariate sensitivity analysis	Threshold not reported Assumed as <€20,000 Euros
Phi et al. (2019); Netherlands (30)	*BRCA1* and *BRCA2* mutation carriers	Microsimulation; Payer; Direct; 1.5%, 4%; 3%; 2017 Euros	LYG; Cost; ICER (cost/LYG)	Univariate sensitivity analysis	Threshold <€20,000 Euros
Taneja et al. (2009); USA (31)	*BRCA1/2* mutation carriers	NR; Healthcare system; Direct; 3%; 2005 US dollars	QALYs; Cost; ICER (cost/QALY gained)	Not sufficient	Threshold not reported Assumed as <$100,000
Pataky et al. (2013); Canada (32)	*BRCA1/2* mutation carriers	Markov Monte Carlo; Health care system; Direct; 3.5%; 2008 CAD dollars	QALYs; Cost; ICER (cost/QALY gained)	One-way, probabilistic sensitivity analysis	Threshold <$50,000–$100,000

Direct cost: screening cost and related procedure, cancer therapy; indirect cost includes cost of not working and loss of productivity.

### Description of Studies: Quality Assessment

Generally, these studies were of good quality (**Supplementary Table S3**), and the main misreported item was conflicts of interest in three studies.

According to the checklist, several items were not mentioned in all selected articles. One study did not present study perspectives, one failed to reveal the model type, and two studies did not mention the time horizon explicitly. Currency details (such as price adjustments) were not shown in one study. In addition, two studies did not state the source of funding. Model parameters, uncertainty, and heterogeneity analyses were not sufficiently considered in one, one, and three studies, respectively. Except for the items above, model information on screening effectiveness and suggested viewpoints for screening strategies were finely described in all manuscripts.

### Outcomes in BRCA1 Mutation Carriers

**Figure 2:**
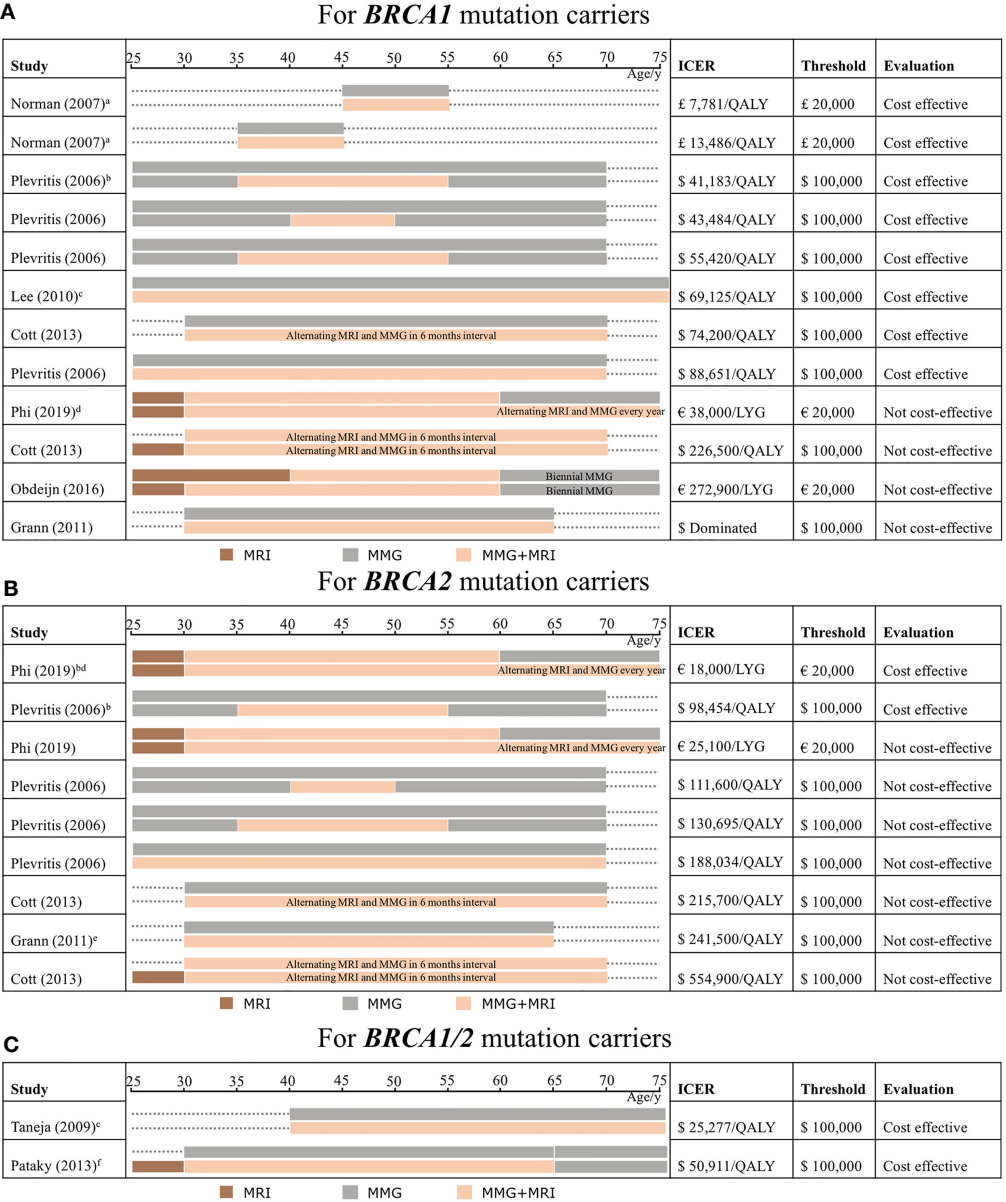
Outcomes from studies from cost-effectiveness analysis of screening strategies comparing mammography and MRI which are categorized by age in *BRCA1* mutation carriers **(A)**, *BRCA2* mutation carriers **(B)**, and *BRCA1/2* mutation carriers **(C)**. The incremental cost-effectiveness ratio (ICER) extracted from our study selection is considered cost-effective if it reaches the threshold. The bars implicate the modality is conducted annually without special illustrations. The explanation of an expensive way which is considered absolutely not cost-effective is discussed. Comparison of the different screening strategies, mainly discussing the combination of MRI and mammography compared with mammography alone (brown color means using MRI alone, gray color means mammography alone, and light orange color means applying a combination of the two). The target population involves *BRCA1* mutation carriers **(A)**, *BRCA2* mutation carriers **(B)**, and not discriminated *BRCA1/2* mutation carriers **(C)**. ICER, threshold, and cost-effectiveness evaluation are shown in each following strategy. MRI, magnetic resonance imaging; MMG, mammography; LYG, life-year gained; QALY, quality-adjusted life years; ICER, incremental cost-effectiveness ratio. a) Screening was conducted for patients with an age range of 10 years and this model involves an age range of women which includes the 30–39 age group and 40–49 age group; b) patients with dense breast; c) screening modalities through a lifetime; d) the result of the cost-effective analysis is under the Dutch discount rate; e) ICER is not reported in the original studies, which is calculated by the average of ICER from its original data; f) the screening modalities continue till 79 years old.

For cost-effectiveness evaluation, the ICER is mainly collected by the comparison of a combination of MRI and mammography with mammography alone. Among *BRCA1* mutation carriers, ICER varies from £7,781 to £13,486 per QALY gained (26) and from $41,183 to $88,651 per QALY gained (25, 27, 28) (**Figure 2A**). Therefore, the most effective screening strategy is combined mammography and MRI annually for *BRCA1* mutation carriers.

Among *BRCA1* mutation carriers, simulated screening regimens are conducted mostly from age 25 or 30 until 70 or 74. One study modeled the screening from a non-specific age range of 10 years, and the surveillance continued for 10 years to compare the difference (26). In particular, dense breast women with breast cancer are considered (25), and ICER is counted under the Dutch discount rate (30). In addition, ICERs exceeding the threshold are distinguished by conducting among old women (30), adding MRI alone compared with no screening from young women (28), and delaying the usage of MRI (29). One dominated ICER (33) is identified with regard to expensive screening strategies.

Considering the starting and ending times of modalities, middle-aged *BRCA1* women who apply the combination of MRI and mammography are mainly discussed in two studies, which revealed that it reaches the minimum level of ICER under the most stringent threshold of $50,000 in the United States and £20,000 in the United Kingdom (25, 26), implying that the combination in age 35 to 54 is cost-effective. In addition, the results also indicate that *BRCA1* women with a prolonged screening strategy to use MRI as an adjunct screening tool roughly from age 30 to 70 (25, 27, 28) are still under a loose cost-effectiveness threshold of $100,000, although ICER rises. Extending the screening until the elderly age from age 60 to 74 will not be cost-effective, whereas the threshold is stringent in the Netherlands (30).

The interval of screening would not affect the cost-effectiveness. Most studies conducted the combination of mammography and MRI annually, whereas one study (28) simulated the alternating modality, with 6-month intervals taking turns to apply MRI and mammography. It seemed that the alternating screening still worked well among *BRCA1* mutation carriers.

### Outcomes in BRCA2 and BRCA1/2 Mutation Carriers

For *BRCA2* mutation carriers, it is only cost-effective using combined mammography and MRI in dense breasts among *BRCA2* mutation carriers (25, 30), as well as measured in the Dutch discount rate (30). In contrast, other studies showed no benefits (25, 28, 30, 33). For the cost-effectiveness evaluation, the ICER above the threshold in these studies varies from €25,100 per LYG (30) and $111,600–$554,900 per QALY gained (25, 28, 33) (**Figure 2B**). *BRCA2* mutation carriers showed higher ICER, which implied that additional MRI with mammography annually may not be cost-effective.

Two studies simulated women not specifically separated into *BRCA1* and *BRCA2* mutation carriers (31, 32). Even so, ICERs were revealed both within the threshold, ranging from $25,277 to $50,911 per QALY gained, when they compared mammography and MRI together with mammography alone (**Figure 2C**).

## Discussion

To date, mortality caused by breast cancer is increasing (34). Patients expect a more effective and cost-effective surveillance strategy to survive (35). According to the guidelines, mammography and MRI are acceptable for *BRCA1/2* mutation and high-risk women. However, the cost-effectiveness of different modalities and their combination is still a topic of controversy (12). Suffering from long-term screening surveillance (8, 12), *BRCA1/2* mutation carriers are supposed to choose a better screening regimen, both for individual perspectives and social resource utility, with consideration of the burden on patients and society (36).

In our systematic review, we found an acceptable ICER using a combination of mammography and MRI among *BRCA1* mutation carriers. ICERs within the threshold of *BRCA1* mutation carriers varied from £7,781 to £13,486 per QALY gained and from $41,183 to $88,651 per QALY gained in four studies. Nevertheless, *BRCA2* mutation carriers benefit less from adjunct MRI, since ICER above the threshold varies from €25,100 per LYG and $111,600–$554,900 per QALY gained in four studies. This implies that mammography alone would be sufficient from a cost-effectiveness perspective for *BRCA2* mutation carriers.

The willingness-to-pay threshold of cost-effectiveness evaluation varies in definition. The WHO demonstrates that the threshold should be less than three times the GDP (gross domestic product) of the country. In the United States, the rigorous level is $50,000, and it has recently expanded to $100,000–$150,000 (37). In the United Kingdom, a threshold below £20,000 is generally acceptable for a cost-effective strategy (38). For the Netherlands, the commonly used value is €20,000 (37). Our selected studies presented the willingness-to-pay threshold, including £20,000 (UK), $50,000–$100,000 (USA and Canada), and €20,000 (Netherlands). We used the given threshold in these studies to analyze the cost-effectiveness.

The timing of combined mammography and MRI regimens in women with *BRCA1* mutations is debatable. When the threshold is strict within £20,000 (UK), $50,000 (USA), and €20,000 (Netherlands), the inclusive ICER within cost-effectiveness evaluation is using the combination of mammography and MRI from age 35 to 54 in *BRCA1* mutation carriers (25, 26). Thus, it is reasonable to suggest that middle-aged women benefit more from adjunct MRI. Considering a loose threshold of $100,000 (USA), the combined mammography and MRI is still cost-effective to apply from age 25–30 to 70–74 (25, 27, 28). However, the elongation of screening time is accompanied by a higher ICER, implying that a prolonged regimen might weaken the benefits of the combination of mammography and MRI.

At younger ages, from 25 to 30, MRI may perform acceptably fine (25, 28, 29, 32) and benefit patients with no radiation harm. Our results showed that postponed mammography is cost-saving (29), which implied that MRI alone from age 25 to 39 might be cost-effective. Concurrently, NCCN and ESMO suggest applying MRI alone from age 25 to 30. In addition, adjunct MRI is coherently cost-effective in middle age (roughly age 35 to 54), and extensive usage of a combination of mammography MRI results in higher ICER, making it less cost-effective. Moreover, extending the combination of MRI and mammography from a younger age will increase potential radiation risks. Accordingly, based on our findings and the guidelines, MRI alone might work well for these young *BRCA1* women.

Considering the elderly age ranging from 60 to 74, mammography alone would work well in cost-effectiveness evaluation since the combination of mammography and MRI exceeded the willingness-to-pay threshold (30). Indirectly, the result suggests that the combination is costly for elderly women with *BRCA1* mutations. Simultaneously, NICE recommends applying mammography alone from age 50 to 69, whereas NCCN and ESMO still propose to use combined mammography and MRI until age 75. Hence, the results indicate that mammography alone might be cost-effective for elderly women. We summarize our findings based on the cost-effectiveness perspective and guidelines in the **Supplementary Figure**, which still needs more studies to test its accuracy and validity.

With regard to *BRCA2* mutation carriers, only dense breast females with breast cancer are reported to economically benefit using combined MRI and mammography (25, 30), which was even provided under restraint scenarios, only from age 60 to 74 in the Dutch discount rate (30).

The difference between *BRCA1* and *BRCA2* could be explained by mutation-related age-specific breast cancer incidence (25, 28) in the sense that *BRCA1* mutation carriers are more likely to develop breast cancer and that cancer is more aggressive. Hence, it would become more beneficial to use MRI for detection to reduce downstream breast cancer treatment and prevent mortality (27), which may balance the high expense of surveillance (28). In addition, false-positive results are likely to appear in *BRCA2* mutation carriers (25). In addition, *BRCA2* mutation carriers tend to develop breast cancer in older age (30). These might result in MRI being less beneficial in *BRCA2* mutation carriers. Therefore, we are more likely to infer that the combination of MRI and mammography for *BRCA1* mutation carriers is more cost-effective than *BRCA2* mutation carriers.

Our study found it most cost-effective using the combination of MRI and mammography in middle-aged *BRCA1* mutation group. Several possible reasons might explain the finding. Cancer detection by adjunct mammography in younger women is low (39–41). It means to detect additional cancer by a combination of MRI and mammography would perform more mammography screening when compared with MRI alone. By increasing the number of mammographies, it results in increased cost, making the combination less cost-effective. Applying mammography in the younger group would endure screening radiation (27, 28). It is still a concern for associated possible risk by radiation-induced cancer (42). The older group also showed fewer cost-effectiveness benefits applying the combination. Increased false-positive findings when performing mammography with adjunct MRI were reported in the older group (43). This might raise the cost for subsequent costs in biopsy and treatment. In addition, declining quality of life and other reasons causing death might explain the reduction of benefits from screening; thus, it leads to a decrease in cost-effectiveness (24). Although other evidence was not given from an economic perspective, mammography with MRI also showed less benefit in younger age among *BRCA1/2* mutation carriers (14, 38). Accordingly, the middle-aged group benefited more from the combination of mammography and adjunct MRI. For young women, we found that MRI alone may be enough to present high sensitivity and no radiation. It is beneficial to conduct MRI from age 25 (38). In addition, a recent RCT study also concluded that MRI could detect early cancer better than mammography (44).

Multiple factors will affect the evaluation of cost-effectiveness. MRI screening is crucial in surveillance, but it is 10 times more expensive than mammography in cost and has restricted resources for patients to gain access (25). Higher false-positive rates leading to unnecessary biopsies should not be ignored (10). Therefore, in our selected studies, confounding factors of cost-effectiveness included screening modality detection sensitivity and specificity (25, 27, 28, 31, 33), false-positive rate (25, 27, 28), breast cancer risk (25, 27, 28, 33), breast cancer mortality (25, 27–29), screening elongating life expectancy (25, 27, 29), and mammography-related radiation (28, 29). The cost of MRI (27, 28, 32, 33), the discount rate (25, 30), and willingness-to-pay threshold (26, 27, 32) associated with payment influence cost-effectiveness. Dense breast women (25, 30), screening interval (28), strategy modification (29), and high-risk women (31) varying in breast cancer prevalence are separately shown to produce an effect on the evaluation of cost-effectiveness.

In this review, we focused on the cost-effectiveness of screening strategies; however, it cannot be considered the only perspective in clinical application. Indeed, a comprehensive evaluation of personal acceptance, resource distribution, modality detection performance, and so forth is needed. With the development of screening modalities, sensitivity and specificity should be updated among *BRCA1/2* mutation carriers and age groups using different screening strategies (10, 43, 45). Additionally, QALYs are difficult to define and it remains a controversial topic to use for analysis (46). False-positive results, anxiety, and potential treatment of detected cancer would influence quality of life, which most studies failed to take into account, which should be considered in the further evaluation of life quality of target women in future analysis.

Several cost-effectiveness analyses were conducted among Asian countries, focusing on high-risk women (47, 48). However, Asian *BRCA1/2* mutation carriers had different characteristics against mutation carriers from Western countries (49). In addition, mainstream screening modalities for Asian high-risk women include ultrasound and mammography, and MRI is rarely used in clinical practice. Our selected studies covered only several developed countries and lacked more evidence from different races.

Moreover, high-risk women are also recommended to apply a combination of MRI and mammography; however, heterogeneous cohorts are difficult to evaluate. Countries vary in the definition of high-risk women that are estimated by different models (50). In the United States, those with lifetime risk above 20% are suggested with adjunct MRI, depending on the risk assessment model, which combines multiple factors, including personal history, prior biopsies, family history, chest radiation, and so forth (17). Studies (31, 51–53) simulated high-risk women classified by different cancer prevalences (31), varied lifetime risks of breast cancer (51), and unknown mutations (52, 53) and analyzed the cost-effectiveness. The outcomes are summarized in **Tables SD1–4** in the **Supplementary Data**. However, the results differ in the definition of high-risk women and screening scenarios, which could not be overlooked due to heterogeneity, making it rather difficult to compare. Therefore, defining and classifying high-risk women is still indefinite so as to draw a conclusion from the present findings, and more evidence is needed to determine the best strategy.

*BRCA1/2* mutation carriers are also associated with increased risk in ovarian cancer, prostate cancer, and pancreatic cancer (54–56). Cost-effectiveness analyses were also conducted. Intensified surveillance, prophylactic and risk-reducing surgery of bilateral mastectomy, and salpingo-oophorectomy were compared from a cost-effective perspective (57, 58). The identification of *BRCA* mutation carriers by genetic testing for early recognition of ovarian cancer was also discussed in cost-effectiveness analysis (58). Furthermore, cost-effectiveness analysis was conducted to discuss applying maintenance olaparib for patients with *BRCA*-mutated metastatic pancreatic cancer (59). Although the cost-effectiveness of prostate cancer screening has been discussed in studies from different nations (60), however, no specific cost evaluation of prostate cancer screening is conducted among *BRCA1/2* mutation carriers. Future attempts and discoveries are expected.

Herein, we only compared *BRCA1/2* mutation carriers because these women are admitted with the greatest lifetime risk. Rated by the CHEERS quality assessment (**Supplementary S3** and **Table SD4** in the **Supplementary Data**), the scores of studies discussing high-risk women were poorer than those of studies focusing on *BRCA1/2* mutation carriers, which might be explained by the uncertainty and heterogeneity of patients. Although only nine studies provided limited evidence, focusing on *BRCA1/2* mutation carriers by deletion of high-risk women, the results were more convincing and integrated.

There are several limitations in our study. First, the selection of inclusive studies was primarily conducted in high-income developed countries, which may influence the final outcomes. In addition, the threshold used by different countries varies in definition, making it hard to compare the available budget, which arises as a relatively unified and objective standard for cost-effectiveness evaluation in the future. Heterogeneity in countries, ethnicity, and publication period among cost-effectiveness analysis results in impairment of evidence strength. Future studies in specific population groups are expected. Additionally, there are different types of mammography, and film screens or digital screens are not considered separate in our study. In addition, although we searched databases and references, it could not cover all studies, with some existing in the gray area. Furthermore, we only target those studies using simulated models evaluating cost-effectiveness. Presently, cost-effectiveness analyses comparing screening modalities in *BRCA1/2* mutation carriers are still limited, failing to cover all age intervals, which requires more investigations to fill the gaps. However, resulting from insufficient evidence to evaluate health benefits and weaknesses from RCTs of screening modality implementation on *BRCA1/2* mutation carriers, simulation model-based economic evaluation can compare the cost-effectiveness of screening tool implementation (20).

## Conclusion

In our systematic review, we found an acceptable ICER using a combination of mammography and MRI among *BRCA1* mutation carriers, especially in middle-aged women. Nevertheless, *BRCA2* mutation carriers benefit less with adjunct MRI, which implies that mammography alone would be enough from a cost-effectiveness perspective. The cost-effectiveness perspective is significant in conducting screening strategies among *BRCA1/2* mutation carriers to surveil breast cancer, which is highly influenced by multiple factors. Recommendations for *BRCA1/2* mutation carriers should take into account cost-effectiveness among different age groups for further clinical usage.

## Data Availability Statement

The datasets presented in this study can be found in online repositories. The names of the repository/repositories and accession number(s) can be found in the article/**Supplementary Material**.

## Author Contributions

JiaxL and ZJ completed the design of the study, conducted the data analysis and interpretation, and wrote the manuscript. MZ completed the design of the study. GL, ZX, XinW, XH, KF, JiangW, and JieW helped with the data collection. JiaqL conceived the study, conducted the data analysis and interpretation, and wrote the manuscript. XiangW conceived the study. All authors revised the manuscript and made the final approval of the manuscript. All authors contributed to the article and approved the submitted version.

## Funding

This research was funded in part by the National Natural Science Foundation of China (81802669 to JiaqL), the CAMS Innovation Fund for Medical Sciences (2020-I2M-C&T-B-068 to JiaqL), the Beijing Hope Run Special Fund (LC2020B05 to JiaqL), the Science and Technology Innovation Foundation for university or college students (202010023021 to JiaxL), and the CAMS Initiative Fund for Medical Sciences (2016-I2M-1-001 to XiangW).

## Conflict of Interest

The authors declare that the research was conducted in the absence of any commercial or financial relationships that could be construed as a potential conflict of interest.

## Publisher’s Note

All claims expressed in this article are solely those of the authors and do not necessarily represent those of their affiliated organizations, or those of the publisher, the editors and the reviewers. Any product that may be evaluated in this article, or claim that may be made by its manufacturer, is not guaranteed or endorsed by the publisher.

## Abbreviations

NCCN, National Comprehensive Cancer Network; NICE, National Institute for Health and Care Excellence; ESMO, European Society for Medical Oncology; LYG, life-years gained; QALY, quality-adjusted life years; ICER, incremental cost-effectiveness ratio; CHEERS, Consolidated Health Economic Evaluation Reporting Standards.
