# Supplementary material for: Cost-Effectiveness Analysis of Imaging Modalities for Breast Cancer Surveillance Among BRCA1/2 Mutation Carriers: A Systematic Review
Source: Front Oncol. 2022 Jan 10;11:763161. doi: 10.3389/fonc.2021.763161 (PMC8785233; doi:10.3389/fonc.2021.763161)
Supplement: Supplementary file 1 [file DataSheet_1.doc]

**Supplementary Data. Cost-effectiveness of high-risk women conducting different screening modalities strategies**

Four studies discussed high-risk women, presenting high lifetime risk of breast cancer, and conducted a simulated cost-effectiveness analysis for screening strategies(1-4). Detailed information about these studies was collected in Table SD1, Table SD2, Table SD3. CHEERS quality assessment was listed in Table SD4. One study simulated indirect cost for loss of productivity and time. Besides, QALY was measured in three studies and LYG in one study. Two studies did not mention the threshold definition. For quality assessment in studies discussing high-risk women, two studies did not present study perspective, and meanwhile the time horizon. One study had a vague description in time horizon. Uncertainty is inadequately discussed in three studies and three studies did not reveal the conflict of interests. Model type and funding information were not reported in one and the other study respectively.

High-risk women were classified by different cancer prevalence (1), the varied lifetime risk of breast cancer (3), and unknown mutation (2, 4). Three selected studies (2-4) added clinical breast examination (CBE) with a combination of mammography to compare with adjunct MRI for cost-effectiveness analysis. The outcomes reported ICER differently, with some (1, 3, 4) declaring cost-effective dropping into threshold whereas some (1, 2, 4) arguing extremely high expanse to perform the combination of mammography and MRI in high-risk women. Results in high-risk women presented an collection of ICER from €21,380 - €33,277 per QALY gained (4), €102,164 - €160,655 per LYG (2) and $45,566 - $310,616 per QALY gained (1, 3). Consequently, higher cancer prevalence increased lifetime risk and distinguished screening modality implementation may result in acceptable ICER and cost-effectiveness conducting combining MRI and mammography. Despite the combination annually (1), alternatingly, or in altered intervals (3), applying MRI alone every 18 months (4) are also proved cost-effective in high-risk women (**Figure SD**).

The cost-effectiveness analysis would be influence by nonnegligible factors, including screening modality detection sensitivity and specificity(1, 2), breast cancer risk(3), cost of MRI(2-4), willing-to-pay threshold and screening intervals(2, 3). High-risk women (1, 3) varying in breast cancer prevalence and familial risk group (2, 4) are separately shown to produce an effect on the evaluation of cost-effectiveness.

**Table SD1. Information of cost-effectiveness analysis studies discussing high-risk women**

| **Study;**  **Author(year)**  **Country/region** | **Target population** | **Model type;**  **Perspective;**  **Type of cost;**  **Discount rate;**  **currency** | **Outcome measures** | **Sensitivity analyses** | **Threshold definition** |
| --- | --- | --- | --- | --- | --- |
| Taneja *et al.* (2009);  US (1) | *BRCA1/2* mutation carriers and high-risk women | NR;  Health care system;  Direct; 3%;  2005 US dollar | QALYs;  Cost;  ICER  (cost/ QALY gained) | Not sufficient | Threshold not reported  Assumed as < $100,000 |
| Saadatmand *et al.* (2013);  Netherlands (2) | High-risk women (15-50% lifetime risk FH+ with no proven mutation) | Microsimulation;  Not reported;  Direct; 3.5%;  Euros (converted into 2013 US dollars) | LYGs;  Cost;  ICER  (cost/LYG)  Cost per detected and treated BC | Sensitivity analysis of key parameters | Threshold not reported  Assumed as < $100,000 |
| Ahern *et al.* (2014);  US (3) | High-risk women (25% lifetime risk) | Monte Carlo Microsimulation;  Not reported;  Direct and indirect; 3%;  2012 US dollars | QALYs;  Cost;  ICER  (cost/QALY gained) | Not sufficient  Scenarios | Cost effectiveness threshold of $100,000 US dollars |
| Geuzinge *et al.* (2020); Netherlands  (4) | High-risk women (20% or more familial risk without known *BRCA1/2* or *TP53*) | microsimulation;  Health care;  Direct; 3%;  2018 Euros | QALYs;  Cost;  ICER  (cost/QALY gained) | One-way and scenarios | Cost effectiveness threshold of €22,000 Euros |

**Table SD2. Screening modalities strategies of cost-effectiveness analysis studies discussing high-risk women**

| **Study;**  **Author(year)**  **Country/region** | **Target population** | **Compared strategies;**  **Initiation age;**  **Time horizon** | **Source of cost data** | **Main conclusion** |
| --- | --- | --- | --- | --- |
| Taneja *et al.* (2009);  US | *BRCA1/2* mutation carriers and high-risk women | 40-75+  Annually MRI+MMG vs annualy MMG  40;  Lifetime | Health center | 1. BRCA1&2 MMG+MRI is cost-effective,  2. high risk depending on prevalence of undiagnosed breast cancer |
| Saadatmand *et al.* (2013);  Netherlands | High-risk women (15-50% lifetime risk FH+ with no proven mutation) | 35-50  annually （MRI+MMG） + CBE every 6 month + vs MMG alone  50-75 biennial MMG  35;  Not reported | Medical Center Institute | High-risk(>15%) women annually (MRI+MMG), that is, the adjunct MRI is very expansive |
| Ahern *et al.* (2014);  US | High-risk women (25% lifetime risk) | 30-74  Alternating yearly MRI and (MMG+CBE)  vs  biennial MRI  30;  NR | Medicare centers and Medicaid services and literature | 25% lifetime-risk stagger MRI+(MMG+CBE) annually from 30-74 is cost-effective, ICER $58,400  50%, 75% lifetime-risk conduct MRI+MMG+CBE (cost decrease + time interval) |
| Geuzinge *et al.* (2020); Netherlands | High-risk women  (lifetime risk: 20% or more) FH+ without known *BRCA1/2* or *TP53*) | 35-60 MRI 18 month interval vs MMG and 60-74 biennial MMG  35;  Lifetime | Netherlands comprehensive cancer organization  And literature | FH+: MRI every 18 month is cost effective in age 35-60 |

**Table SD3. Outcomes for high-risk group in screening management cost-effectiveness analysis**

| **Outcomes for BRCA1/2 mutation carriers and high-risk group in screening management cost-effectiveness analysis** | | | | | | |
| --- | --- | --- | --- | --- | --- | --- |
| **Study** | **Screening method** | **Cost per patient** | **Health benefits per patient** | **Comparison group cost per patient** | **Comparison group health benefits per patient** | **ICERs** |
| Taneja *et al.* (2009) | ***Cost-effective***  High-risk women  Annually MRI + MMG  vs  MMG from 40-lifetime  (3% prevalence) | $22,390 | QALYs 206337 | $13,049 | QALYs 20.6132 | $45.566 |
|  | ***Not cost-effective***  High-risk women  Annually MRI + MMG  vs  MMG from 40-lifetime (0.5% prevalence) | $29,870 | QALYs 206473 | $13,579 | QALYs 20.6439 | $310,616 |
| Saadatmand *et al.* (2013) | ***Not cost-effective***  High-risk women  Annual MRI+MMG+every 6 month CBE from 35-50  vs  No screening between 35-60  Both 50-75 biennial MMG | $10,258.897 | Extra LYG 0.195 | 2,378.880 | 0 | $134,932 (€ 102,164) |
|  | ***Not cost-effective***  High-risk women  alternating biennial MRI and MMG+every 6 month CBE  vs  annual MMG + CBE  both 50-75 biennial MMG | $6345.618 | Extra LYG 0.166 | $4669.372 | Extra LYG 0.139 | $212,183 (€160,655 ) |
| Ahern *et al.* (2014) | ***Cost-effective***  High-risk women (25% lifetime risk)  Stagger yearly MRI+(MMG+CBE) from 30-74  vs  MRI biennial from 30-74 | $37,900 | QALYs 53.5448 | $36,500 | QALY 53.5215 | $58,400 |
|  | ***Scenario***  High-risk women (50% lifetime risk)  * 70% reduction of MRI cost  Stagger MRI+(MMG+CBE) every 6 months from 30-74  vs  MRI annually from 30-74 | $53,500 | QALYs 53.2531 | NR | QALYs 53.2139 | $84,400 |
|  | ***Scenario***  High-risk women (75% lifetime risk)  Biennial MRI + (MMG+CBE) every 6 months from 30-74  vs  Stagger yearly MRI+(MMG+CBE) from 30-74 | $79,900 | QALYs 52.8321 | NR | QALYs 52.7572 | $62,800 |
| Geuzinge *et al.* (2020) | ***Not cost-effective***  High-risk women  From 35-60  annual MRI +annual CBE +biennial MMG  **vs**  annual MMG + annual CBE  both 60-75 biennial MMG | €9742.033 | QALYs 22.964 | €7084.767 | QALYs 22.885 | €33,277 |
|  | ***Cost-effective***  High-risk women  MRI every 18 Month from 35-60  **vs**  annual MMG from 35-60  both 60-75 biennial MMG | €6,896.883 | QALYs 22.939 | €6,306.999 | QALYs 22.912 | €21,380 |

**Table SD4. CHEERS checklist for quality assessment of studies discussing high-risk women**

| **Item No.** | **Item** | **Recommendation** | Taneja *et al.* (2009);  US | Saadatmand *et al.*  (2013);  Netherlands | Ahern *et al.*  (2014);  US | Geuzinge *et al.* (2020);  Netherlands |
| --- | --- | --- | --- | --- | --- | --- |
| 1 | Title | Economic study or CEA or describe comparison of intervention | Y | Y | Y | Y |
| 2 | Abstract | Structured summary | Y | Y | Y | Y |
| 3 | Background and objectives | Context of the study question | Y | Y | Y | Y |
| 4 | Target population and subgroups | Characteristics and reason | Y | Y | Y | Y |
| 5 | Setting and location | State relevant aspects | Y | Y | P | Y |
| 6 | Study perspective | Perspective and cost evaluated | Y | NR | NR | Y |
| 7 | Comparators | Intervention compared and reason | Y | Y | Y | Y |
| 8 | Time horizon | Time and why appropriate | P | NR | NR | Y |
| 9 | Discount rate | Used for costs and outcomes | Y | Y | Y | Y |
| 10 | Choice of health outcomes | Measure of benefit | Y | Y | Y | Y |
| 11 | Measurement of effectiveness | Describe the design and effectiveness data | Y | Y | Y | Y |
| 12 | Measurement and valuation of preference-based outcomes | Population and methods used to elicit preferences for outcomes | Y | Y | Y | Y |
| 13 | Estimating resources and costs | Resource item and unit cost | Y | Y | Y | Y |
| 14 | Currency, price date, and conversion | Costs as reported | Y | Y | Y | Y |
| 15 | Choice of model | Model type | NR | Y | Y | Y |
| 16 | Assumptions | Assumptions under this model | Y | Y | Y | Y |
| 17 | Analytical methods | All analytical methods supporting evaluation | Y | Y | Y | Y |
| 18 | Study parameters | Input value | Y | Y | Y | Y |
| 19 | Incremental costs and outcomes | Cost, ICER | Y | Y | Y | Y |
| 20 | Characterizing uncertainty | Results of uncertainty related to the structure and assumption | P | P | P | Y |
| 21 | Characterizing heterogeneity | Subgroup of patients influencing the results | Y | Y | Y | P |
| 22 | Study finding, limitations, generalizability, and current knowledge | Conclusion and limitation | Y | Y | Y | Y |
| 23 | Source of funding | Funding and related role | NR | Y | Y | Y |
| 24 | Conflicts of interest | Conflicts of interests | NR | NR | NR | Y |
| Total | Score |  | 20 | 20.5 | 20 | 23.5 |


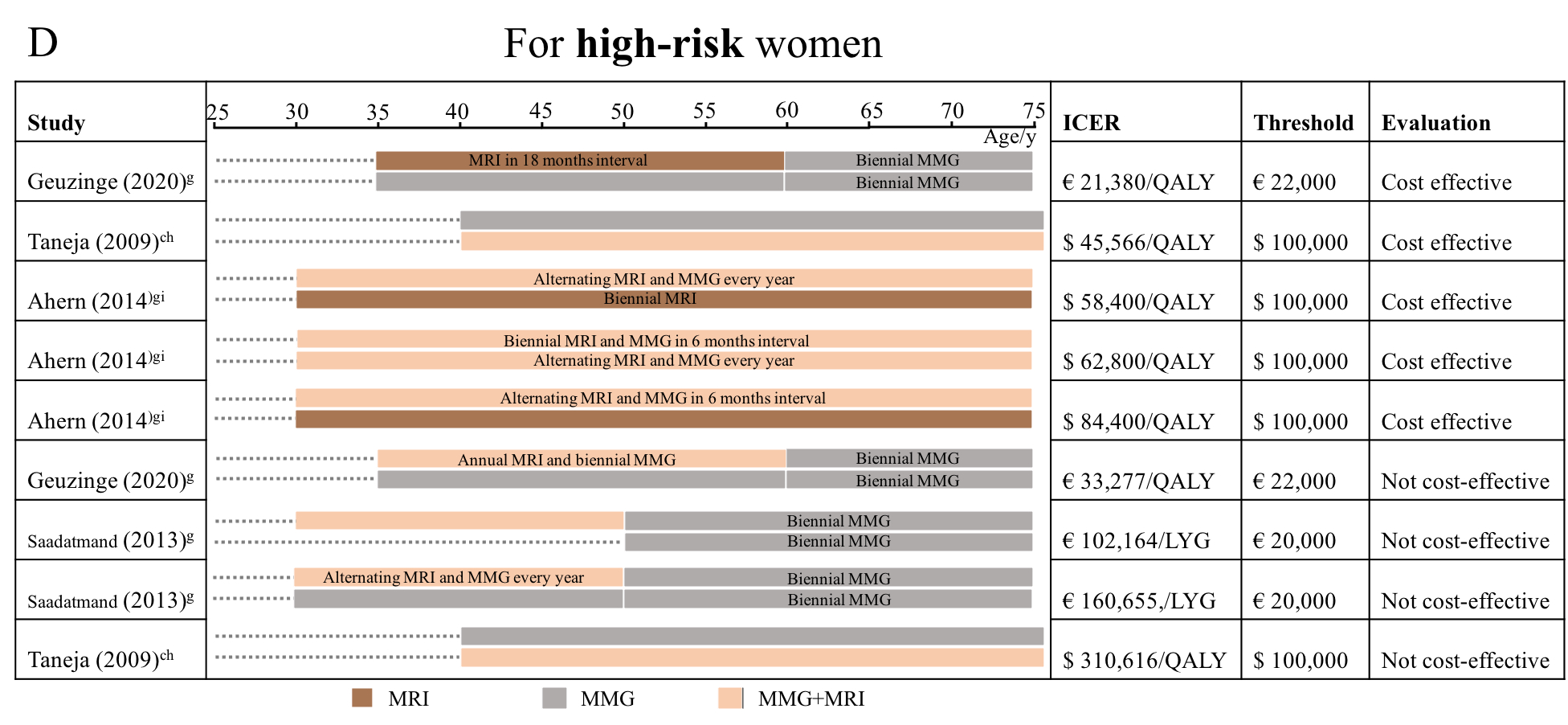


**Figure SD. Outcomes from studies from cost-effectiveness analysis of screening strategies comparing mammography and MRI which are categorized by age in high-risk women**

The incremental cost-effectiveness ratio (ICER) extracted from our study selection is considered cost-effective if it reaches the threshold. The bars implicate the modality is conducted annually without special illustrations. Dominated is the explanation of an expensive way which is considered absolute not cost-effective. Figure 2D compared different screening strategies, mainly discussing a combination of MRI and mammography comparing with mammography alone (Brown color means using MRI alone, gray color mammography alone and light orange color applying a combination of the two). The target population is high-risk women (Fig 2D). ICER, threshold, and cost-effectiveness evaluation are shown in each following strategy.

(MRI: Magnetic resonance imaging, MMG: mammography, LYG: life-year gained, QALY: quality-adjusted life years, ICER: incremental cost-effectiveness ratio)

c. the screening modalities round through lifetime

g. the screening modalities is accompanied with clinical breast examination (CBE)

h. different in prevalence

i. different in lifetime risk

**References:**

1. Taneja C, Edelsberg J, Weycker D, Guo A, Oster G, Weinreb J. Cost effectiveness of breast cancer screening with contrast-enhanced MRI in high-risk women. J Am Coll Radiol. 2009;6(3):171-9.

2. Saadatmand S, Tilanus-Linthorst MM, Rutgers EJ, Hoogerbrugge N, Oosterwijk JC, Tollenaar RA, et al. Cost-effectiveness of screening women with familial risk for breast cancer with magnetic resonance imaging. J Natl Cancer Inst. 2013;105(17):1314-21.

3. Ahern CH, Shih YC, Dong W, Parmigiani G, Shen Y. Cost-effectiveness of alternative strategies for integrating MRI into breast cancer screening for women at high risk. Br J Cancer. 2014;111(8):1542-51.

4. Geuzinge HA, Obdeijn I-M, Rutgers EJT, Saadatmand S, Mann RM, Oosterwijk JC, et al. Cost-effectiveness of Breast Cancer Screening With Magnetic Resonance Imaging for Women at Familial Risk. JAMA oncology. 2020.
