# Supplementary material for: Cost-Effectiveness Analysis of Imaging Modalities for Breast Cancer Surveillance Among BRCA1/2 Mutation Carriers: A Systematic Review
Source: Front Oncol. 2022 Jan 10;11:763161. doi: 10.3389/fonc.2021.763161 (PMC8785233; doi:10.3389/fonc.2021.763161)
Supplement: Supplementary file 2 [file DataSheet_2.doc]

**Supplementary Figure. Summarization of existing evidence and recommendation to conduct screening strategies from cost-effectiveness perspective for each age group in *BRCA1*-carriers (A) and *BRCA2*-carriers (B)**


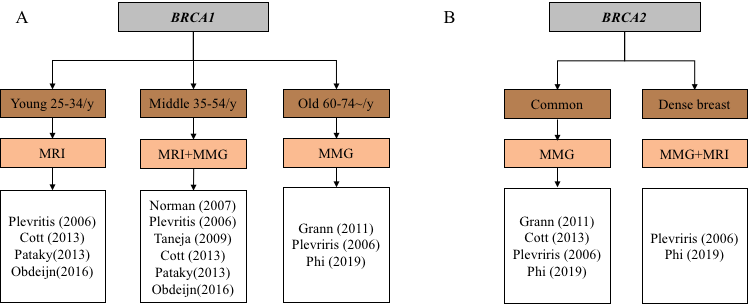


(MRI: Magnetic resonance imaging, MMG: mammography)

*BRCA1*-mutation carriers are more likely to benefit from the combination of MMG and MRI, especially cost-effective in middle age women in *BRCA1*-mutation carriers. The age range is not an accurate range which is inferred from our selection of studies. *BRCA2*-mutation carriers present fewer benefits from the adjunct MRI, which implies MMG may be cost-effective enough. Dense breast *BRCA2*-mutation carriers could be suggested using the combined MRI and MMG.
