# Supplementary material for: Cost-Effectiveness Analysis of Imaging Modalities for Breast Cancer Surveillance Among BRCA1/2 Mutation Carriers: A Systematic Review
Source: Front Oncol. 2022 Jan 10;11:763161. doi: 10.3389/fonc.2021.763161 (PMC8785233; doi:10.3389/fonc.2021.763161)
Supplement: Supplementary file 3 [file Table_1.doc]

**Supplementary Table S1. Search strategy**

| **MEDLINE**  (Through  PubMed) | #1 (brca1 gene[MeSH Terms]) OR (brca2 gene[MeSH Terms])   **6,402 results**  #2 ((((((((((brca1 gene[MeSH Terms]) ) OR (brca2 gene[MeSH Terms])) OR (familial[Title/Abstract])) OR (hereditary[Title/Abstract])) OR (inherited[Title/Abstract])) OR (high risk[Title/Abstract])) OR (BRCA[Title/Abstract])) OR (BRCA 1[Title/Abstract])) OR (BRCA 2[Title/Abstract])) OR (BRCA 1/2[Title/Abstract])    **531,158 results**  #3 breast neoplasm[MeSH Terms]   **293,646 results**  #4(((breast neoplasm[MeSH Terms]) OR (breast cancer[Title/Abstract])) OR (breast carcinoma[Title/Abstract])) OR (breast tumor[Title/Abstract])      **378,736 results**  #5 #2 AND #4  **20,782 results**  #6 cost effectiveness[MeSH Terms] **81,706 results**  #7 ((((((((((cost effectiveness[MeSH Terms]) OR (economic evaluation[Title/Abstract])) OR (economic analy*[Title/Abstract])) OR (economic stud*[Title/Abstract])) OR (decision analy*[Title/Abstract])) OR (decision tree[Title/Abstract])) OR (decision model[Title/Abstract])) OR (cost-effectiveness[Title/Abstract])) OR (cost-benefit[Title/Abstract])) OR (cost-utility[Title/Abstract])) OR (cost-analy*[Title/Abstract]) **140,472 results**  #8 #5 AND #7 **328 results**  #9 Mass Screening[MeSH Terms] **128,707 results**  #10 ((((Mass Screening[MeSH Terms]) OR (mammography[MeSH Terms])) OR (mammogra*[Title/Abstract])) OR (MRI[Title/Abstract])) OR (Magnetic Resonance Imaging[Title/Abstract]) **539,329 results**  #11 #8 AND #10 **103 results**  #12 #11 AND ("1990/01/01"[Date - Publication] : "3000"[Date - Publication])  **101 results** |
| --- | --- |
| **EMBASE** | #1 brca1/de OR brca2/de **34,572 results**  #2 familial:ab,ti OR hereditary:ab,ti OR inherited:ab,ti OR 'high risk':ab,ti OR brca:ab,ti **768,675 results**  #3 brca1:ab,ti OR brca2:ab,ti **26,625 results**  #4 #1 OR #2 OR #3 **789,375 results**  #5 'breast neoplasm’/de **1,869 results**  #6 'breast cancer':ab,ti OR 'breast carcinoma':ab,ti OR 'breast tumor':ab,ti **417,664 results**  #7 #5 or #6 **418,166 results**  #8 #4 and #7 **33,255 results**  #9 ‘cost effectiveness’/de **152,741 results**  #10 'economic evaluation':ab,ti OR 'economic analy$':ab,ti OR 'economic stud$':ab,ti **13,512 results**  #11 'decision tree':ab,ti OR 'decision model':ab,ti  **13,623 results**  #12 'cost effectiveness':ab,ti OR 'cost-benefit':ab,ti OR 'cost-utility':ab,ti OR 'cost-analy$':ab,ti **100,325 results**  #13 #9 or #10 or #11 or #12**203,925 results**  #14 #8 and #13 **515 results**  #15 ‘mass screening’/de **59,277 results**  #16 ‘mammography’/de **49,925 results**  #17 'mammogra$':ab,ti OR 'mri':ab,ti OR 'magnetic resonance imaging':ab,ti  **573,270 results**  #18 #15 or #16 or #17 **670,162 results**  #19 #14 and #18**140 results**  #20 #19 AND [1990-2020]/py **140 results** |
| **The Cochrane Library** | #1  MeSH descriptor: [Genes, BRCA1] explode all trees 82  #2 MeSH descriptor: [Genes, BRCA2] explode all trees 67  #3 #1 or #2 82  #4 ((familial or hereditary or inherited or (high risk) or brca or brca1 or brca2 or brca1/2)):ti,ab,kw 89093  #5 #3 or #4 89093  #6 MeSH descriptor: [Breast Neoplasms] explode all trees 12949  #7 ('breast cancer' or 'breast carcinoma' or 'breast tumor’):ti, ab,kw 36529  #8 #6 or #7 37165  #9 #5 and #8 3868  #10 MeSH descriptor: [CosT-Benefit Analysis] explode all trees  6898  #11 ('economic evaluation' or 'economic analy*' or 'economic stud*' or 'decision analy*' or 'decision tree' or 'decision model' or 'cost-effectiveness' or 'cost-benefit' or 'cost-utility' or 'cost-analy*’):ti, ab,kw 45767  #12 #10 or #11 45767  #13 #9 and #12 266  #14 MeSH descriptor: [Mass Screening] explode all trees 3733  #15 MeSH descriptor: [Mammography] explode all trees 774  #16 (mammogra* or MRI or 'magnetic resonance imaging’):ti, ab,kw 34412  #17 #14 or #15 or #16 37835  #18 #13 and #17 **29 results** |
| **Scopus** | # 1( TITLE-ABS-KEY ( "BRCA" OR "BRCA1" OR "BRCA2" OR "BRCA1/2" ) OR TITLE-ABS-KEY ( "familial" OR "hereditary" OR "inherited" OR "high risk" ) ) 976,591 document results  #2 TITLE-ABS-KEY ( "Breast neoplasms" OR "breast cancer" OR "breast carcinoma" OR "breast tumor" ) 536,708 document results  #3 #1 and #2 40,846 document results  #4 ( TITLE-ABS-KEY ( "economic evaluation" OR "economic analy*" OR "economic stud*" ) OR TITLE-ABS-KEY ( "decision analy*" OR "decision tree" OR "decision model" ) OR TITLE-ABS-KEY ( "cost effectiveness" OR "cost-effectiveness" OR "cost-benefit" OR "cost-utility" OR "cost-analy*" ) ) 674,158 document results  #5 #3 and #5 1,004 document results  #6 TITLE-ABS-KEY ( "mass screening" OR "mammography" OR "mammogra*" OR "MRI" OR "magnetic resonance imaging" ) 1,047,740 document results  #7 #5 and #6 320 document results  # #7 AND ( EXCLUDE ( PUBYEAR , 1988 ) OR EXCLUDE ( PUBYEAR , 1986 ) OR EXCLUDE ( PUBYEAR , 1982 ) OR EXCLUDE ( PUBYEAR , 1978 ) OR EXCLUDE ( PUBYEAR , 1976 ) OR EXCLUDE ( PUBYEAR , 1974 ) ) **311 document results** |
| Web of Science | #1 TS=(BRCA OR BRCA1 OR BRCA2 OR BRCA1/2 OR Familial or hereditary or inherited or (high risk) ) 2431964  #2 TS= ((Breast neoplasms) OR (breast cancer) OR (breast carcinoma) OR (breast tumor) ) 970292  #3 #1 and #2 95724  #4 TS=( (economic evaluation) OR (economic analy*) OR (economic stud*) OR (decision analy*) OR (decision tree) OR (decision model ) OR (cost effectiveness) OR (cost-effectiveness) OR (cost-benefit) OR (cost-utility) OR (cost-analy*) ) 2362814  #5 #3 and #4 4569  #6 TS=((mass screening) OR (mammography) OR (mammogra*) OR MRI OR (magnetic resonance imaging) ) 1260197  #7 #5 and #6 858  #8 #7 Refined by: [excluding] PUBLICATION YEARS: ( 1989 OR 1988 OR 1987 OR 1986 OR 1985 OR 1984 OR 1982 OR 1981 OR 1978 OR 1976 )  **841** |
