# Supplementary material for: Cost-Effectiveness Analysis of Imaging Modalities for Breast Cancer Surveillance Among BRCA1/2 Mutation Carriers: A Systematic Review
Source: Front Oncol. 2022 Jan 10;11:763161. doi: 10.3389/fonc.2021.763161 (PMC8785233; doi:10.3389/fonc.2021.763161)
Supplement: Supplementary file 4 [file Table_2.doc]

**Supplementary Table S2. Information of inclusive cost-effectiveness analysis studies**

| **Study;**  **Author(year)**  **Country/region** | **Target population** | **Compared strategies;**  **Initiation age;**  **Time horizon** | **Model type;**  **Perspective;**  **Type of cost;**  **Discount rate;**  **Currency** | **Source of cost data** | **Outcome measures** | **Sensitivity analyses** | **Main conclusion** | **threshold definition** |
| --- | --- | --- | --- | --- | --- | --- | --- | --- |
| Plevritis *et al.* (2006);  US | *BRCA1* &  *BRCA2*  mutation  carriers | 25-69  Annual MMG+MRI vs  MMG  Strategies vary in starting and stopping time  25 or other starting time;  Lifetime | Continuous time Monte Carlo;  Societal;  Direct & cost of time lost from work;  3%;  2005 US dollars | Published literature and medicare payments | QALYs  Cost;  ICER (cost/QALY gained) | One-way,  multivariate | 1. MRI more effective for *BRCA1* than *BRCA2*  2 cost-effectiveness of MRI varies by age | Cost effectiveness threshold of $100,000 US dollars |
| Norman *et al.* (2007);  UK | *BRCA1*  Mutation  carriers | 30-39 & 40-49 aged women for 10 years surveillance  Annual MRI+MMG vs  MMG  Conducting for 10 years;  30-39 & 40-49;  Lifetime | Markov;  National health service;  Direct;  3.5%;  2006 UK pounds | NHS and Personal social services | QALY;  Cost;  ICER(cost/QALY gained) | Univariate sensitivity analysis and probabilistic sensitivity analysis | The addition of MRI with mammography in 40-49 and 30-39 aged women conducting 10-year screening is cost effective. 40-49 lower ICER implying more cost0effective | Cost effectiveness threshold of £20,000 pounds |
| Lee *et al.* (2010);  US | *BRCA1*  mutation  carriers | 25- till ending annually MRI+MMG  vs  MMG;  25;  Lifetime | Markov Monte Carlo;  Societal;  Direct;  3%;  2007 US dollars | Medicare reimbursement rates  And literature | QALY;  Cost;  ICER(cost/ QALY gained) | Univariate sensitivity analysis and multi-variate sensitivity analysis | Annual combined MMG and MRI is cost-effective | Cost effectiveness threshold of $50,000-100,000 US dollars |
| Grann *et al.* (2011);  US | *BRCA1* & *BRCA2*  mutation  carriers | 30-65  Annually MRI+MMG  vs  MMG;  30;  Lifetime | Markov Monte Carlo;  Societal;  Direct and indirect;  3%;  2009 US dollars | Centers for medicare and medicaid services | QALY;  Cost;  ICER(cost/QALY gained) | Probabilistic sensitivity analysis | Annual combined MMG + MRI very expensive and costly | Threshold not reported  Assumed as < $100,000 |
| Cott *et al.* (2013);  US | *BRCA1* & *BRCA2*  mutation  carriers | 25 or 30 till ending MRI+MMG  (or 25-29 MRI alone)  with interval of 6 month or annually  vs  MMG alone;  25 or 30;  Lifetime | Markov Monte Carlo;  Not mentioned of perspective;  Direct and indirect;  3%;  2010 US dollars | Medicare physician fee schedule | QALY  Cost;  ICER (cost/QALY gained) | Univariate sensitivity analysis, Two-way,  multi-parameter sensitivity analysis | 1.Alternating MMG+MRI from 30 is cost-effective.  2.In *BRCA1* carriers, it is more cost-effective than *BRCA2*. | Threshold not reported  Assumed as < $100,000 |
| Obdeijn *et al.* (2016); Netherlands | *BRCA1*  mutation carriers | Target in 30-39y  MRI 25-60+MMG 40-60 annual +MMG 60-74 biennial vs MRI 25-60 + MMG 30-60 +MMG 60-74 biennial;  30 or 40 for MMG;  lifetime | Microsimulation;  Health care system;  Direct;  3.5%;  not clearly mentioned Euros | Hospitals, national breast cancer screening program and national guidelines in 2012 | LYG;  Cost;  ICER(cost/LYG) | Univariate sensitivity analysis | Delaying MMG to 40 years increase breast cancer deaths, decrease of effectiveness, but avoid radiation risk deaths more cost saving | Threshold not reported  Assumed as < €20,000 Euros |
| Phi *et al.* (2019); Netherlands | *BRCA1* & *BRCA2*  mutation  carriers | 60-74  Annually MRI from 25-29 and 30-59 MRI+MMG  Alternating annual MRI+MMG vs annual MMG to only dense breast in 60-74;  60 for comparison;  Lifetime | Microsimulation;  Payer;  Direct;  1.5,4%;3%;  2017 Euros | Literature | LYG;  Cost;  ICER(cost/LYG) | Univariate sensitivity analysis | 1.*BRCA1*: not cost-effective;  2.*BRCA2* with dense breast : alternating annual MRI +MMG is cost-effective using Dutch discount rate | Threshold < €20,000 Euros |
| Taneja *et al.* (2009);  US | *BRCA1/2* mutation carriers and high-risk women | 40-75+  Annually MRI+MMG vs annualy MMG  40;  Lifetime | NR;  Health care system;  Direct;  3%;  2005 US dollars | Health center | QALYs;  Cost;  ICER(cost/ QALY gained) | Not sufficient | 1. *BRCA1/2* MMG+MRI is cost-effective,  2. high risk depending on prevalence of undiagnosed breast cancer | Threshold not reported  Assumed as < $100,000 |
| Pataky *et al.* (2013);  Canada | *BRCA1/2* mutation carriers | 30-64  Annually MRI+MMG vs MMG;  (MRI+MMG: 25-29 MRI; MMG alone 65-79  MMG: 65-79)  25;  Lifetime | Markov Monte Carlo;  Health care system;  Direct;  3.5%;  2008 CAD dollars | Medical service commission fee schedule | QALYs;  Cost;  ICER(cost/QALY gained) | One-way, probabilistic sensitivity analysis | *BRCA1/2* MRI+MMG annually compared with MMG alone is cost-effective $50,900/QALY  It is influenced by threshold. | Threshold < $50,000-$100,000 |

Direct cost: screening cost and related procedure, cancer therapy
