# Supplementary material for: Cost-Effectiveness Analysis of Imaging Modalities for Breast Cancer Surveillance Among BRCA1/2 Mutation Carriers: A Systematic Review
Source: Front Oncol. 2022 Jan 10;11:763161. doi: 10.3389/fonc.2021.763161 (PMC8785233; doi:10.3389/fonc.2021.763161)
Supplement: Supplementary file 5 [file Table_3.doc]

**Supplementary Table S3. CHEERS checklist for quality assessment**

| **Item No.** | **Item** | **Recommendation** | Plevritis *et al.* (2006);  US | Norman *et al.* (2007);  UK | Lee *et al.* (2010);  US | Grann *et al.* (2011);  US | Cott *et al.* (2013);  US | Obdeijn *et al.* (2016); Netherlands | Phi *et al.*  (2019); Netherlands | Taneja *et al.* (2009);  US | Pataky *et al.* (2013);  Canada |
| --- | --- | --- | --- | --- | --- | --- | --- | --- | --- | --- | --- |
| 1 | Title | Economic study or CEA or describe comparison of intervention | Y | Y | Y | P | Y | P | Y | Y | Y |
| 2 | Abstract | Structured summary | Y | Y | Y | Y | Y | Y | Y | Y | Y |
| 3 | Background and objectives | Context of the study question | Y | Y | Y | Y | Y | Y | Y | Y | Y |
| 4 | Target population and subgroups | Characteristics and reason | Y | Y | Y | Y | Y | Y | Y | Y | Y |
| 5 | Setting and location | State relevant aspects | Y | Y | Y | Y | Y | Y | Y | Y | Y |
| 6 | Study perspective | Perspective and cost evaluated | Y | Y | Y | Y | NR | Y | Y | Y | Y |
| 7 | Comparators | Intervention compared and reason | Y | Y | Y | Y | Y | Y | Y | Y | Y |
| 8 | Time horizon | Time and why appropriate | Y | P | Y | Y | Y | Y | Y | P | Y |
| 9 | Discount rate | Used for costs and outcomes | Y | Y | Y | Y | Y | Y | Y | Y | Y |
| 10 | Choice of health outcomes | Measure of benefit | Y | Y | Y | Y | Y | Y | Y | Y | Y |
| 11 | Measurement of effectiveness | Describe the design and effectiveness data | Y | Y | Y | Y | Y | Y | Y | Y | Y |
| 12 | Measurement and valuation of preference-based outcomes | Population and methods used to elicit preferences for outcomes | Y | Y | Y | Y | Y | Y | Y | Y | Y |
| 13 | Estimating resources and costs | Resource item and unit cost | Y | Y | Y | Y | Y | Y | Y | Y | Y |
| 14 | Currency, price date, and conversion | Costs as reported | Y | Y | Y | Y | Y | NR | Y | Y | Y |
| 15 | Choice of model | Model type | Y | Y | Y | Y | Y | Y | Y | NR | Y |
| 16 | Assumptions | Assumptions under this model | Y | P | Y | Y | Y | Y | Y | Y | Y |
| 17 | Analytical methods | All analytical methods supporting evaluation | Y | Y | Y | Y | Y | Y | Y | Y | Y |
| 18 | Study parameters | Input value | Y | P | Y | Y | Y | Y | Y | Y | Y |
| 19 | Incremental costs and outcomes | Cost, ICER | Y | Y | Y | Y | Y | Y | Y | Y | Y |
| 20 | Characterizing uncertainty | Results of uncertainty related to the structure and assumption | Y | Y | Y | Y | Y | Y | Y | P | Y |
| 21 | Characterizing heterogeneity | Subgroup of patients influencing the results | Y | P | P | Y | Y | Y | Y | Y | P |
| 22 | Study finding, limitations, generalizability, and current knowledge | Conclusion and limitation | Y | Y | Y | Y | Y | Y | Y | Y | Y |
| 23 | Source of funding | Funding and related role | Y | Y | Y | Y | Y | NR | Y | NR | Y |
| 24 | Conflicts of interest | Conflicts of interests | Y | NR | Y | NR | Y | Y | Y | NR | Y |
| Total | Score |  | 24 | 21 | 23.5 | 22.5 | 23 | 21.5 | 24 | 20 | 23.5 |

Y: reported completely (score: 1), P: reported partially (score: 0.5), N: not reported (score: 0),
