# Supplementary material for: Cost-Effectiveness Analysis of Imaging Modalities for Breast Cancer Surveillance Among BRCA1/2 Mutation Carriers: A Systematic Review
Source: Front Oncol. 2022 Jan 10;11:763161. doi: 10.3389/fonc.2021.763161 (PMC8785233; doi:10.3389/fonc.2021.763161)
Supplement: Supplementary file 6 [file Table_4.doc]

**Supplementary Table S4. Detailed data of inclusive cost-effectiveness analysis studies**

| **Outcomes for *BRCA1* mutation carriers in screening management cost-effectiveness analysis** | | | | | | |
| --- | --- | --- | --- | --- | --- | --- |
| **study** | **Screening method** | **Cost per patient** | **Health benefits per patient** | **Comparison group cost per patient** | **Comparison group health benefits per patient** | **ICERs** |
| Plevritis *et al.* (2006) | ***Cost-effective (most beneficial)***  Annual MMG from 25-69+adjunct annual MRI from 40-49  vs  25-69 annual MMG only | $66,145 | QALYs 21.710 | $59,826 | QALYs 21.565 | $43,484 |
|  | ***Cost-effective***  annual MMG from 25-69+ adjunct annual MRI from 35–54  vs  annual from 25-69 MMG only | $72,512 | QALYs 21.794 | $59,826 | QALYs 21.565 | $55,420 |
|  | ***Cost-effective in extremely dense breast***  Annual MMG from 25-69+ annual adjunct MRI from 35-54  Vs  Annual MMG from 25-69 | NR | NR | $59,826 | QALYs 21.565 | $41,183 |
|  | ***Cost-effective***  Annual MMG + MRI from 25-69  vs  annual MMG 25-69 | $87,147 | QALYs 21.873 | $59,826 | QALYs 21.565 | $88,651 |
| Norman *et al.* (2007) | ***Cost-effective in 30-39 years old women***  Annual MMG+MRI  vs  MMG for 10-year surveillance | £7,638 | QALYs 18.427 | £5,392 | QALYs 18.260 | £13,486 |
|  | ***Cost-effective in 40-49 years old women***  Annual MMG+MRI  vs  MMG for 10-year surveillance | £8,840 | QALYs 16.418 | £6,590 | QALYs 16.129 | £7,781 |
| Lee *et al.* (2010) | ***Cost-effective***  Annual MMG+MRI  vs  MMG from 25 till lifetime | $110,973 | QALYs 44.624 | $100,336 | QALYs 44.46 | $69,125 |
|  | ***Not cost-effective***  Annual MRI  vs  MMG from 25 till lifetime | $108.641 | QALYs 44.50 | $100,336 | QALYs 44.46 | Dominated |
| Grann *et al.* (2011) | ***Not cost-effective***  Without knowing of high-risk  Annual MMG+MRI from 30-64  vs  Annual MMG from 30-64 | $192,429 | QALYs 18.08 | $179,629 | QALYs 18.08 | Dominated |
|  | ***Not cost-effective***  With knowing of high-risk  Annual MMG+MRI from 30-64  vs  Annual MMG from 30-64 | $192,418 | QALYs 18.66 | $179,617 | QALYs 18.55 | **$107,571-$126,742***  **(average$117,156)** |
| Cott *et al.* (2013) | ***Cost-effective***  Alternating MMG+MRI (6 months interval) from 30-70  vs  annual MMG from 30-70 | $118,000 | QALYs 44.37 | $109,000 | QALYs 44.25 | $74,200 |
|  | ***Not cost-effective***  25-29 MRI + Alternating annually MMG+MRI from 30-70 (6 months interval)  vs  Alternating annually MMG+MRI from 30-70 (6 months interval) | $121,700 | QALYs 44.39 | $110,300 | QALYs 44.37 | $226,500 |
| Phi *et al.* (2019) | ***Not cost-effective using Dutch discount rate***  25-29 MRI+ 30-59 MMG+MRI + Alternating annual MRI +MMG in 60-75  Vs  Annual MMG from 60-75 | Additional cost €6,428 | Additional LYG  0.169 | NR | NR | €38,000 |
|  | ***Not cost-effective using international discount rate***  25-29 MRI+ 30-59 MMG+MRI + Alternating annual MRI +MMG in 60-75  Vs  Annual MMG from 60-75 | Additional cost €6,816 | Additional LYG  0.132 | NR | NR | €51,600 |
| Obdeijn *et al.* (2016) | ***Modified strategy more cost-saving***  Annual MRI from 25-60+ annual MMG from 40-60+ MMG from 60-74 biennial [modified strategy]  vs  Annual MRI from 25-60+annual MMG from 30-60 +MMG from 60-74 biennial | €10,664.21 | LYG 22.7076 | €11,210.01 | LYG 22.7096 | €272,900 |

| **Outcomes for *BRCA2* mutation carriers in screening management cost-effectiveness analysis** | | | | | | |
| --- | --- | --- | --- | --- | --- | --- |
| **Study** | **Screening method** | **Cost per patient** | **Health benefits per patient** | **Comparison group cost per patient** | **Comparison group health benefits per patient** | **ICERs** |
| Plevritis *et al.* (2006) | ***Cost-effective in extremely dense breast***  Annual MMG from 25-69+ annual adjunct MRI from 35-54  Vs  Annual MMG from 25-69 | NR | NR | 31989 | QALYs 23.431 | $98,454 |
|  | ***Not cost-effective***  annual MMG from 25-69+ adjunct annual MRI from 35–54  vs  annual from 25-69 MMG only | $45,682 | QALYs 23.536 | $31,989 | QALYs 23.431 | $130,695 |
|  | ***Not cost-effective***  Annual MMG from 25-69+adjunct annual MRI from 40-49  vs  25-69 annual MMG only | $38,806 | QALYs 23.492 | $31,989 | QALYs 23.431 | $111,600 |
|  | ***Not cost-effective***  Annual MMG + MRI from 25-69  vs  annual MMG 25-69 | $61,594 | QALYs 23.589 | $31,989 | QALYs 23.431 | $188,034 |
| Grann *et al.* (2011) | ***Not cost-effective***  Without knowing of high-risk  Annual MMG+MRI from 30-64  vs  Annual MMG from 30-64 | $177,918 | QALYs 18.49 | $165,843 | QALYs 18.44 | **$204,661-$294,512***  **(average$241,500)** |
|  | ***Not cost-effective***  With knowing of high-risk  Annual MMG+MRI from 30-64  vs  Annual MMG from 30-64 | $177,934 | QALYs 19.12 | $165,760 | QALYs 18.94 | **$64,413-$71,193***  **(average$67,633)** |
| Cott *et al.* (2013) | ***Not cost-effective***  Alternating MMG+MRI (6 months interval) from 30-70  vs  annual MMG from 30-70 (6 months interval) | $115,000 | QALYs 45.58 | $102,100 | QALYs 45.52 | $215,700 |
|  | ***Not cost-effective***  25-29 MRI + Alternating annually MMG+MRI from 30-70 (6 months interval)  vs  Alternating annually MMG+MRI from 30-70 (6 months interval) | $118,800 | QALYs 45.59 | $115,000 | QALYs 45.58 | $554,900 |
| Phi *et al.* (2019) | ***Cost-effective in dense breast women using Dutch discount rate***  25-29 MRI+ 30-59 MMG+MRI + Alternating annual MRI +MMG in 60-75  Vs  Annual MMG from 60-75 | Additional cost €5,206 | Additional LYG 0.290 | NR | NR | €18,000 |
|  | ***Not cost-effective in dense breast women using international discount rate***  25-29 MRI+ 30-59 MMG+MRI + Alternating annual MRI +MMG in 60-75  Vs  Annual MMG from 60-75 | Additional cost €5,490 | Additional LYG 0.219 | NR | NR | €25,100 |

| **Outcomes for *BRCA1/2* mutation carriers in screening management cost-effectiveness analysis** | | | | | | |
| --- | --- | --- | --- | --- | --- | --- |
| **Study** | **Screening method** | **Cost per patient** | **Health benefits per patient** | **Comparison group cost per patient** | **Comparison group health benefits per patient** | **ICERs** |
| Taneja *et al.* (2009) | ***Cost-effective***  *BRCA1/2*  Annually MRI + MMG  vs  MMG from 40-lifetime | $27,605 | QALYs 206113 | $20,578 | QALYs 20.5835 | $25,277 |
| Pataky *et al.* (2013) | ***Cost-effective***  *BRCA1/2*  Annually MRI + MMG (25-29 MRI only)  vs  MMG both from 30-64 + 65-79 both MMG only | $9,893 | QALYs 22.66 | $5,201 | QALYs 22.57 | $50,911 |

MMG: mammography; MRI, magnetic resonance imaging; LYG, life years gained, QALYs, quality-adjusted life years; ICER, incremental cost-effectiveness ratio; NR: not reported

All strategies involve costs from original currencies are not converted to the same currency.
